# Packing topology in crystals of proteins and small molecules: a comparison

**DOI:** 10.1038/s41598-017-12699-4

**Published:** 2017-10-16

**Authors:** Oliviero Carugo, Olga A. Blatova, Elena O. Medrish, Vladislav A. Blatov, Davide M. Proserpio

**Affiliations:** 1 Department of Chemistry, University of Pavia, viale Taramelli 12, I-27100 Pavia, Italy; 2 Department of Structural and Computational Biology, University of Vienna, Campus Vienna Biocenter 5, A-1030 Vienna, Austria; 3 Samara Center for Theoretical Materials Science (SCTMS), Samara University, Ac. Pavlov St. 1, Samara, 443011 Russia; 4 School of Materials Science and Engineering, Northwestern Polytechnical University, Xi’an, Shaanxi 710072 People’s Republic of China; 5 Università degli Studi di Milano, Dipartimento di Chimica, Via C. Golgi 19, 20133 Milano, Italy

## Abstract

We compared the topologies of protein and small molecule crystals, which have many common features – both are molecular crystals with intermolecular interactions much weaker than intramolecular interactions. They also have different features – a considerably large fraction of the volume of protein crystals is occupied by liquid water while no room is available to other molecules in small molecule crystals. We analyzed the overall and local topology and performed multilevel topological analyses (with the software package ToposPro) of carefully selected high quality sets of protein and small molecule crystal structures. Given the suboptimal packing of protein crystals, which is due the special shape and size of proteins, it would be reasonable to expect that the topology of protein crystals is different from the topology of small molecule crystals. Surprisingly, we discovered that these two types of crystalline compounds have strikingly similar topologies. This might suggest that molecular crystal formations share symmetry rules independent of molecular dimension.

## Introduction

According to McPherson^1^, the first observation of a protein crystal - earthworm hemoglobin - is about 150 years old^2^. However, during several decades, protein crystallization was just a technique for protein purification from complex extracts. Universal interest in protein crystallization arose stunningly only one century later, when Kendrew and Perutz proved that it was possible to determine crystal structures of proteins (sperm whale myoglobin and then hemoglobin)^3–5^. Thereinafter, thousands of protein crystals have been produced and about 120,000 protein crystal structures are now available in the Protein Data Bank^6,7^.

Protein crystallization is considered the bottleneck of protein crystallography. Proteins are extremely soluble, in physiological conditions, and an “evolutionary negative design” has been proposed to have determined the difficulty to crystallize proteins^8^. However, protein crystallization *in vivo* has been observed and it allowed the determination of the first protein crystal structure *in vivo* ^9^.

Although they are crucial in macromolecular crystallography, protein crystals attracted only little attention in recent years. Few studies have been dedicated to the analysis of protein-protein interactions at crystal contacts and their comparison with physiological interactions^10–12^. Later, a survey on the frequency of crystal packing interactions appeared^13^. The importance of packing bridges – small molecules or metal cations that bridge two adjacent and symmetry related macromolecules – in protein crystallization was also discovered^14,15^.

Protein crystals are suboptimally packed molecular crystals, because of their special shape and size. They contain a substantial number of cavities and channels filled by liquid water. It is believed that, roughly, 20–80% of the crystal volume is made by liquid water^16–18^. This is the reason why enzymes can catalyze chemical reactions in the solid state^19,20^ and why protein crystals can be soaked to prepare heavy atom derivatives that can be used to solve the phase problem^21^. Therefore, beside water molecules that strongly interact with the protein molecules^22–24^, protein crystals contain a considerable amount of aqueous solution, containing several types of molecules that were present in the crystallization cocktail. Consequently, crystallization free energy of proteins is very modest, only 3–6 kcal/mole, relative to the dissolved state in solution^25^.

The topology of packing in protein crystals may be different from that of other molecular solids, where the packing is, in general, optimal and where there are no internal cavities filled with amorphous material. It is thus interesting to compare the topology of small organic and protein crystals. The most widespread model of molecular crystals is the Kitaigorodskii’s model^26^, according to which each molecule in a *homomolecular* crystal tends to be surrounded by 12 other molecules; other coordination numbers (10 or 14) occur much rarer. However, a comprehensive treatment of all molecular structures stored in the Cambridge Structural Database using a computer-realized Voronoi approach^27,28^ showed that coordination number 14 is more abundant than 12. This was explained by the model of deformable molecules, in contrast to the model of rigid ones used by Kitaigorodskii.

Since the approach used in references^27,28^ can be applied to the molecules of any size and shape it was natural to use it for comparative analysis of packings of protein and small molecules.

## Experimental

### Data selection

#### Protein data

All protein crystal structures were taken from the Protein Data Bank^6,7^, according to the following criteria. We discarded structures containing nucleic acids and structures with an average B-factor smaller than 10 Å^2^ or larger than 40 Å^2^. We considered only structures with 50–500 amino acids in the asymmetric unit, with resolution better than 3 Å, with working R-factor better than 0.25, and determined at 80–120 K. We discarded structures deposited without their experimental diffraction data. Redundancy was reduced to 30% sequence identity. Structures with missing protein atoms or with protein atoms deposited with zero occupancy were discarded as well as the structures where non-protein and non-water atoms are more than 5% of the total number of atoms.

We note that these are standard criteria for protein crystal structure selection from the Protein Data Bank^29,30^. In particular, resolution and R-factor thresholds ensure that unreliable structures are excluded from the analyzed data sets; moreover, we considered a homogeneous set containing only low temperature crystal structures, which are now the routine, since packing might depend on temperature; furthermore, limitations on the average B-factor and on protein dimension ensure that anomalous structures – much larger or smaller or much more or less flexible than customary proteins – are removed from the data sets; and eventually, redundancy reduction to 30% sequence identity, though very crude, is necessary to avoid distribution biases.

It is also important to observe that the exclusion of structures containing too many (more than 5%) hetero-atoms ensures that crystal contacts due to the presence of co-crystallized small molecules (the so called packing bridges) are minimized. Analogously, the exclusion of incomplete structures, where some of the protein atoms/residues were undetected, ensures that no protein-protein crystal packing contacts are neglected.

We prepared three ensembles of protein crystal structures. In the first, we collected only monomeric proteins that crystallized with only one molecule per asymmetric unit (*monomer* set; 394 structures). In the second, we grouped dimeric proteins that crystallized with only one dimer per asymmetric unit (*dimer* set; 207 structures). In the third and last ensemble, we pulled together structures of monomeric proteins with two molecules in the asymmetric unit (*double* set; 164 structures).

These data sets might seem small if compared to the number of entries deposited in the Protein Data Bank. However, it is necessary to consider that only 31% of the PDB files contain monomeric proteins with one molecule per asymmetric unit; only 7% contain dimeric proteins with one molecule per asymmetric unit; and only 9% contain monomeric proteins with two molecules per asymmetric unit. Moreover, redundancy reduction to 30% maximal sequence identity implies a 6-fold decrease of the number of PDB entries. Eventually, in the large majority of the protein crystal structures deposited in the Protein Data Bank there are residues that have not been seen experimentally^31^. Since they are usually at the protein surface, it is not possible to use these structures to perform reliable analyses of the crystal packing. We note also that the quality of the data extracted from the Protein Data Bank is becoming a crucial issue in structural bioinformatics^29,32^. It is also important to observe that although these are high quality and non-redundant datasets of protein crystal structures, it is impossible to estimate the extent to which they represent the entire protein “universe”, a considerable fraction of which is constituted, for example, by membrane proteins that are under-represented in the Protein Data Bank^33^.

The identification codes of all these crystal structures are listed in the Supplementary Information (Table S1).

#### Small molecules data

Crystallographic data for organic molecular crystals consisting of chemically equivalent molecules were taken from the CSD (release 5.38). We have not analyzed molecular crystals of coordination or organometallic compounds, which have different chemical nature compared to proteins. In total, we have considered all 166,297 structures, which contain one independent molecule and 10,240 structures with two independent chemically equivalent molecules in the asymmetric unit. The entries containing incomplete, erroneous data, or disordered structures as well as those with R_f_ > 10% were excluded; no other restrictions were applied.

### Crystal contacts identification

#### Protein data

The crystal contacts were identified as described previously^13^. The asymmetric unit was transformed according to all symmetry operations and translated up to three times along the axes *a*, *b*, and *c* in both negative and positive directions. Two molecules – one, the reference molecule, is in the asymmetric unit and the other, the satellite, is symmetry related to the first – were considered to be in contact if at least one atom of one of them is closer than 4.5 Å from an atom of the other one. We refer to this method below as ‘Distance’ method.

#### Small molecules data

All interatomic contacts were determined by means of the ‘Domains’ method^34^ implemented into the ToposPro package^35^. This method is based on the Voronoi partition and accounts not only for interatomic distances, but also for atomic surrounding and screening effects. An interatomic contact A-B is assumed to exist if there is a face of an atomic Voronoi polyhedron with a solid angle, Ω > 1.5% of the total solid angle 4π steradian and the line A-B crosses the face (the contact is *direct*). To distinguish valence and non-valence contacts a set of additional criteria was used as described in reference^34^. The ‘Domains’ method is designed to process large samples of crystal structures in an automated mode, which was crucial for our study. At the same time, this method was never used for proteins; thus, it was important to check if this method gives similar results for proteins as the ‘Distance’ method.

**Figure 1:**
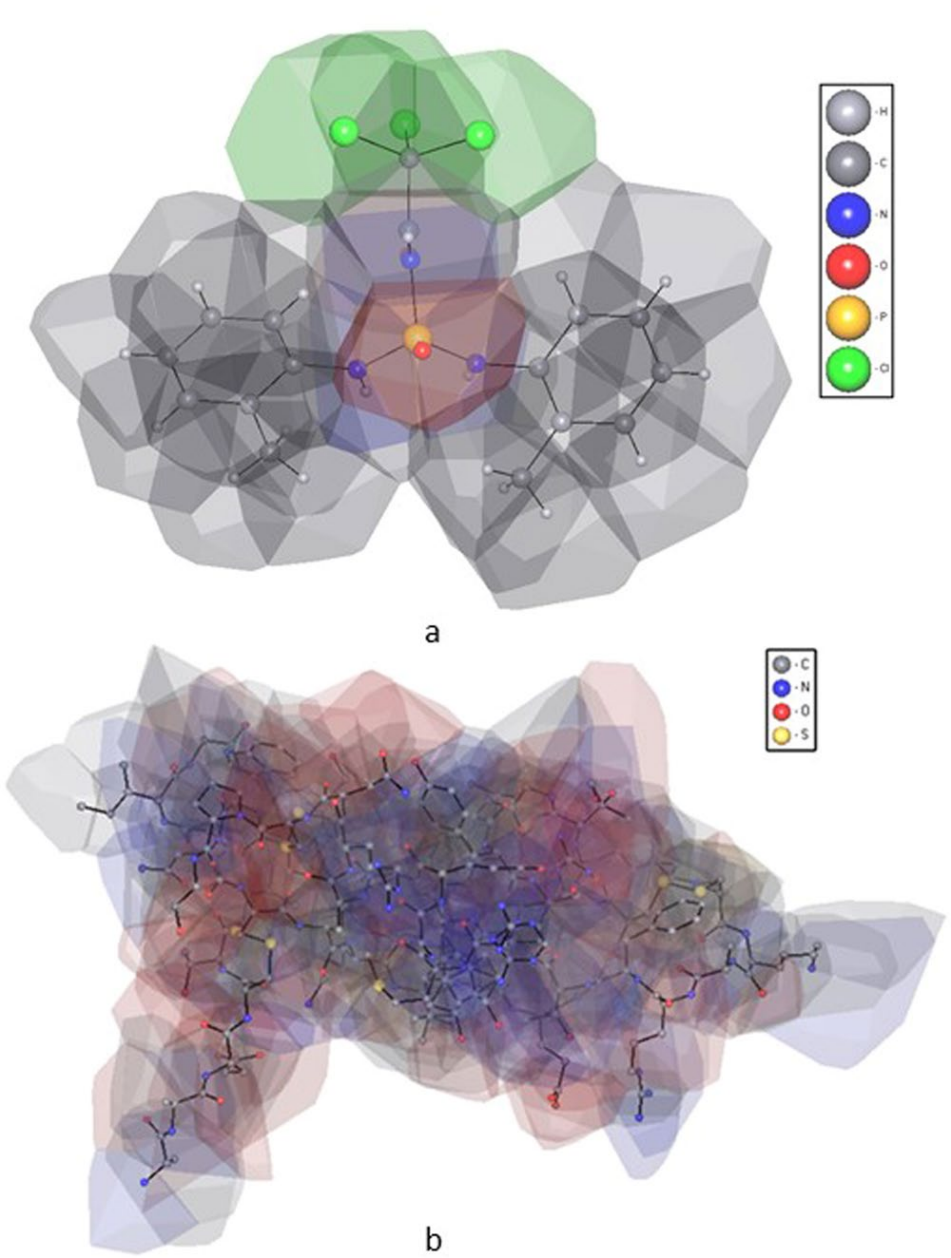
Molecular Voronoi polyhedron of: (**a**) N,N′-Bis(2-methylphenyl)-N′′-(2,2,2-trichloroacetyl)phosphoric triamide (ABAQAX)^47^; (**b**) tick inhibitor of human tryptase (PDB entry 2uux)^48^.

The molecular coordination number was computed for the molecular Voronoi polyhedron, which is a union of Voronoi polyhedra of all atoms of the molecule (Fig. 1)^27,28^.

**Figure 2:**
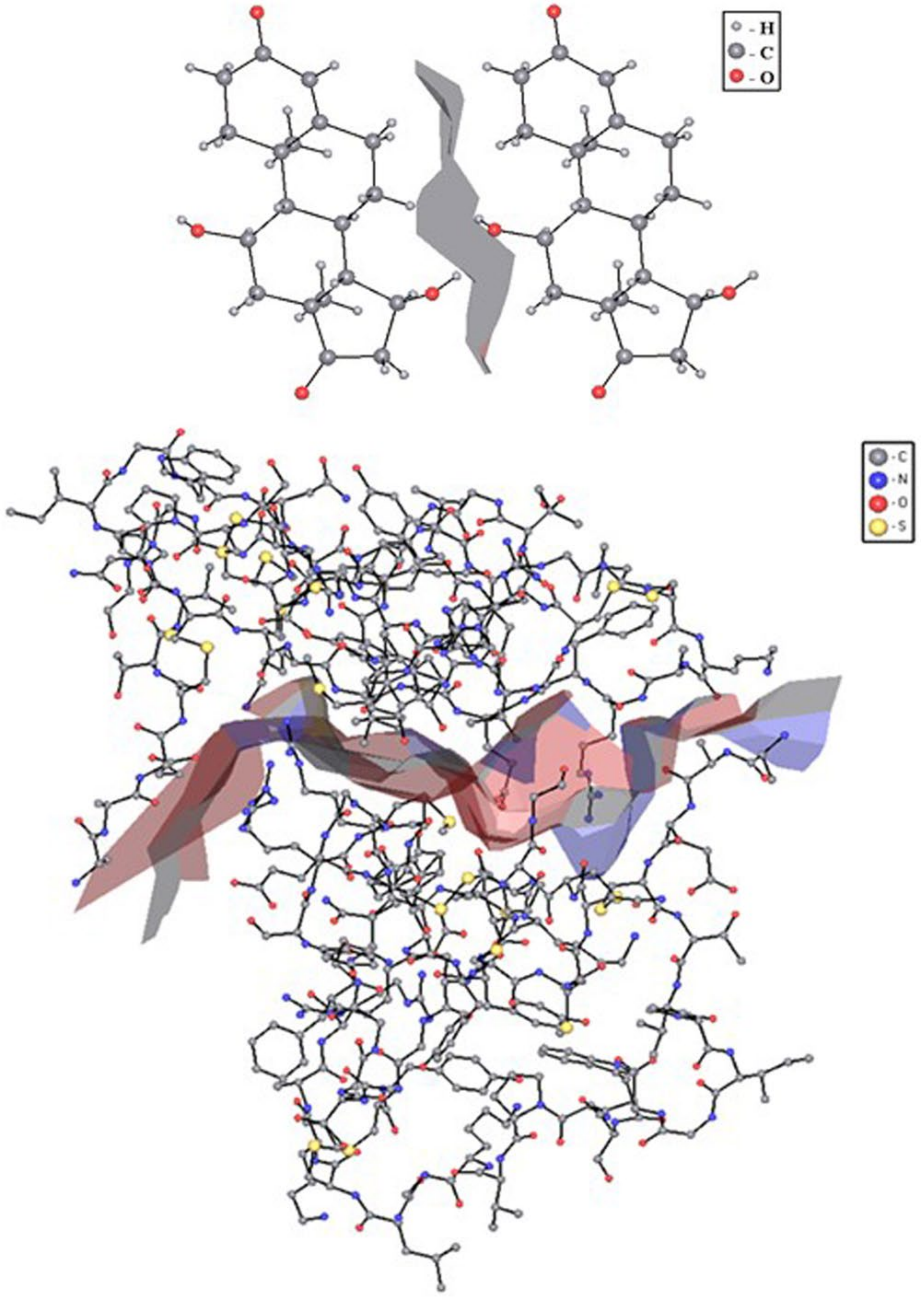
Two molecules of: (**a**) 11α,15α -Dihydroxyandrost-4-ene-3,17 (ABAPEA)^49^; (**b**) two molecules of the tick inhibitor of human tryptase (PDB entry 2uux)^48^, separated by a surface, combined from the faces of their Voronoi polyhedra, which correspond to the intermolecular contacts.

External faces of the molecular Voronoi polyhedron correspond to intermolecular contacts, and two molecules are considered interacting if there is at least one intermolecular contact between them (Fig. 2)^27^.

This method was also used to compute coordination numbers of some protein molecules for comparison with the simple geometrical approach described above.

### Topological analysis

**Figure 3:**
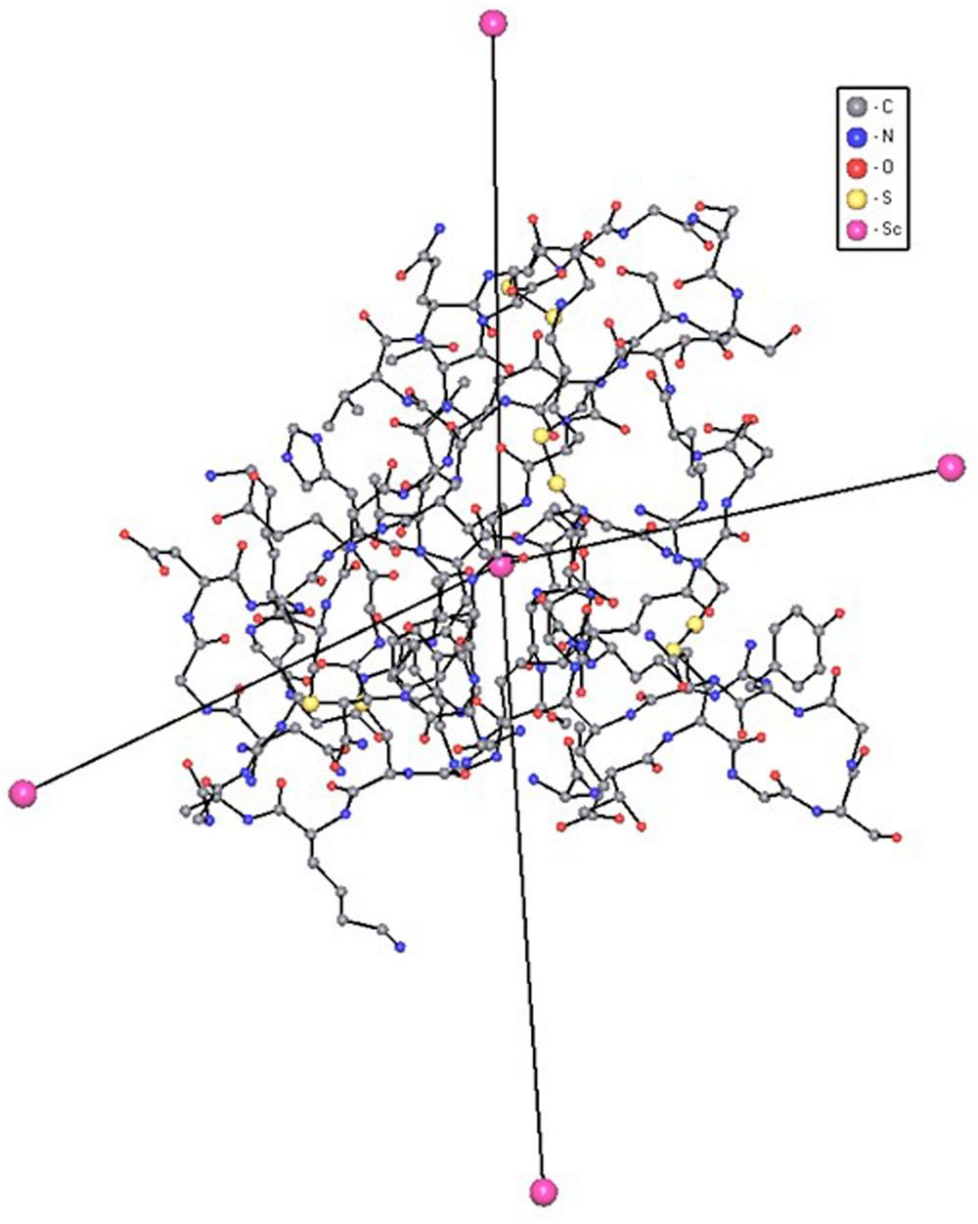
A molecule of the bubble protein 1uoy^50^, its center of mass and centers of mass of four neighboring molecules (pink balls), which represent a fragment of the underlying net.

To analyze molecular packings of both proteins and small molecules we squeezed each molecule into its center of gravity to obtain the so-called *underlying net*, *i.e*. a net, which keeps the information on the connection of molecules throughout the crystal, and ignores the information about their geometrical properties and internal structure (Fig. 3)^36^. As a result, the crystal structure is represented as an infinite graph, whose vertices and edges correspond to molecules and intermolecular links, respectively. Such simplification of the structure allows us to put aside differences in size, shape and other geometrical features of molecular ensembles and to focus on the method of their local and overall connection, i.e. on the crystal architecture as a whole. This is a way to find correlations between packings of so different objects as small molecules and proteins.

The packings represented by the same underlying net are topologically equal and belong to the same *isoreticular series*, which is designated by a topological type symbol. There are several nomenclatures for topological types; here we use two of them: the RCSR three-letter codes^37^ and the *N*D*n* symbols used in the collection of ToposPro topological databases (TTD)^38^, where *N* is the series of coordination numbers for all independent nodes, D is one of the letters C, L or T, indicating the dimensionality of the net (C, L, T for chain, layer, and three-periodic framework, respectively) and *n* is the number of the net with a given ND sequence in the TTD collection. For example, the 16T4 symbol means a three-periodic net with 16-coordinated nodes (i.e. any molecule has 16 neighbors in the packing) and it is the fourth 16-coordinated net in the TTD collection.

Since the strength of intermolecular contacts is quite different, the topology of a molecular packing strongly depends on which contacts are taken into account. Ignoring interactions of a particular level of strength, we generate different underlying nets for the same crystal structure. Each underlying net specifies the packing topology at a given level of interaction and corresponds to a structure *representation*.

**Figure 4:**
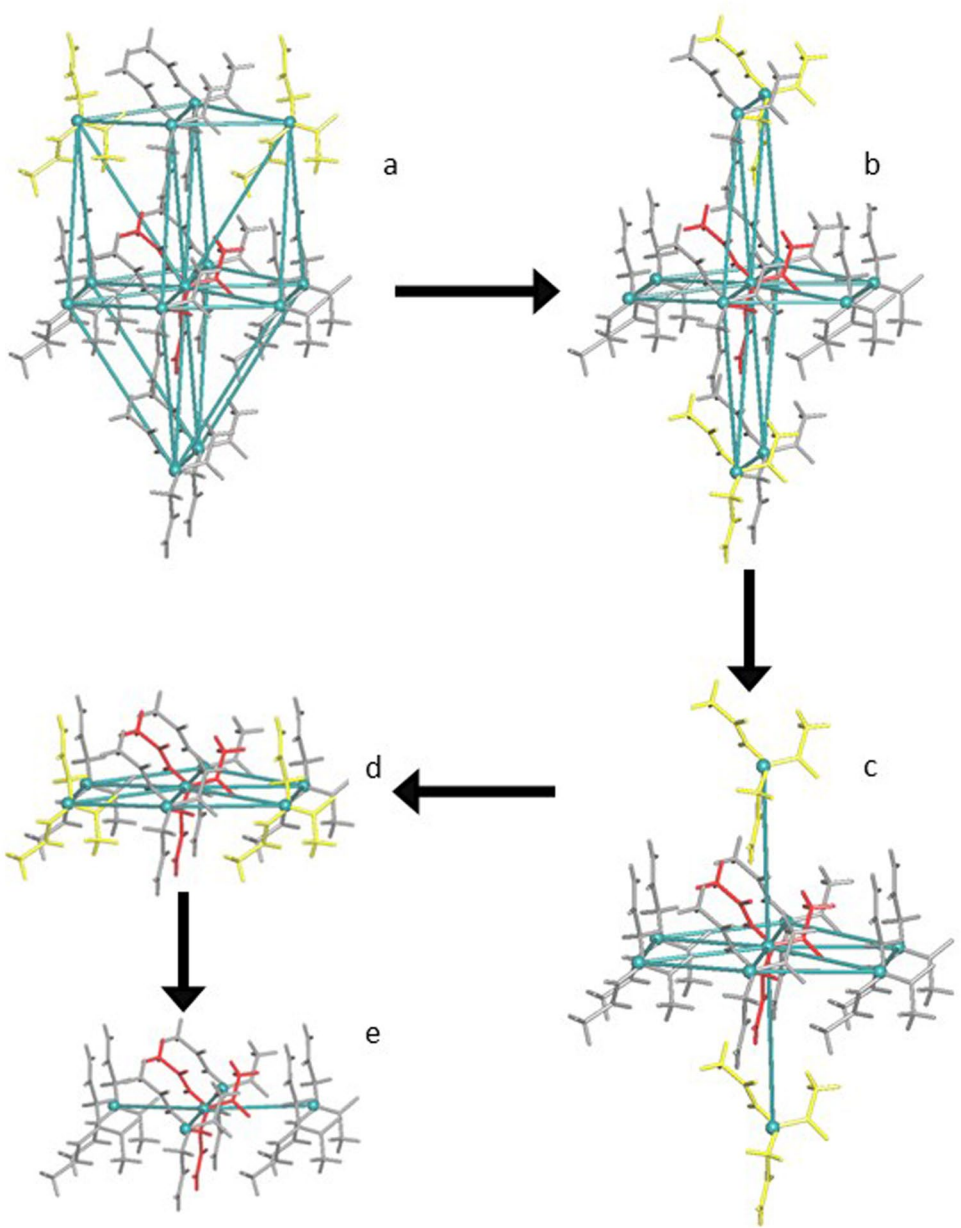
An illustration of the multilevel topological analysis for the 2-(1,2-diacetylhydrazino)acetamide (ABELAW)^51^: (**a**) 12 coordinated underlying net, **hcp** topology; (**b**) 10 coordinated underlying net, **bct** topology; (**c**) 8 coordinated underlying net, **hex** topology; (**d**) 6 coordinated underlying net, **hxl** topology; (**e**) 4 coordinated underlying net, **sql** topology. The origin molecule is in red, the molecules, connected at different levels of interactions are in yellow.

For small molecule crystals, we estimated the strength of an intermolecular interaction by the value of Ω_mol_ that is the part of the sum of solid angles of all contacts, which are formed by a given molecule; this part corresponds to all contacts between a given pair of molecules. ToposPro includes a special procedure, which generates all possible representations by subsequently breaking groups of weak intermolecular contacts with close Ω_mol_ (the difference between neighboring Ω_mol_ in the group does not exceed 1.5% of the total solid angle 4π steradian). As a result the structure is considered at different levels of intermolecular interactions. The underlying net was constructed for each level of intermolecular interaction to obtain the information about the way of molecular connection. Such a *multilevel* analysis, which we successfully used for elucidation of organic molecular crystals^39^, allows one to separate underlying motifs, which determine the structure architecture. These motifs can be the same in the molecular packings, which differ in details of weaker intermolecular contacts (Fig. 4). In our study we considered only the representations with molecular coordination numbers ≤14; there are 105,549 molecular packings with such connectivity among the 166,297 structures studied. The topologies of molecular packings with a higher connectivity are non-specific and can be usually reduced to a known topology with a smaller coordination number after discarding weak intermolecular contacts.

For protein crystals, strengths of crystal packing contacts were estimated based on their dissociation free energies as described below.

### Crystal contacts thermodynamics

Since protein crystallization occurs in aqueous solutions and crystals are largely impregnated by aqueous solutions, the formation and stability of crystal packing contacts cannot be considered out of solvent context. For this reason, we used the program PISA^40^ to estimate the stability of individual crystal packing contacts formed by the reference molecule and each of its satellites, which were generated by applying all the symmetry operations to the asymmetric unit and by applying three translations along all the axes in the negative and in the positive directions. Intermolecular contacts were defined as pairs of atoms, one in the reference molecule and the other in a satellite molecule, closer than 4.5 Å.

According to the PISA definitions, the dissociation free energy *ΔG*_*diss*_ is defined as:

1 $${\rm{\Delta }}{G}_{diss}=-{\rm{\Delta }}{G}_{int}-T\cdot {\rm{\Delta }}S$$

where *ΔG*_*int*_ is the binding free energy of the two molecules (the reference molecule plus one of its satellites), T is the absolute temperature, and ΔS is the entropy change upon association of the two molecules. If ΔG_diss_ > 0, the crystal packing contact is thermodynamically unstable in solution and is present only in the crystal as an experimental artifact. Δ*G*_*int*_ is defined as:

2 $${\rm{\Delta }}{G}_{int}={\rm{\Delta }}{G}_{solv(AB)}-{\rm{\Delta }}{G}_{solv(A)}-{\rm{\Delta }}{G}_{solv(B)}+{\rm{\Delta }}{G}_{cont(AB)}+{\rm{\Delta }}{G}_{es(AB)}$$

where *ΔG*_*solv(AB)*_ is the solvation energy of the dimeric entity AB (A is the reference molecule and B is one of its satellites), *ΔG*_*solv(A)*_ is the solvation energy of the reference molecule, *ΔG*_*solv(B)*_ is the solvation energy of the satellite molecule, *ΔG*_*cont(AB)*_ is the contact energy between molecules A and B, and *ΔG*_*es(AB)*_ is the electrostatic energy between molecules A and B. Krissinel and Henrick developed a strategy to compute all these components necessary to estimate the *ΔG*_*diss*_ values. This strategy, in part, is based on heuristic guesses of some terms, for example for the atomic solvation parameter, the hydrogen bond, salt bridge, and disulfide bond contributions^40^.

## Results and Discussion

### Overall topologies of molecular packings

**Figure 5:**
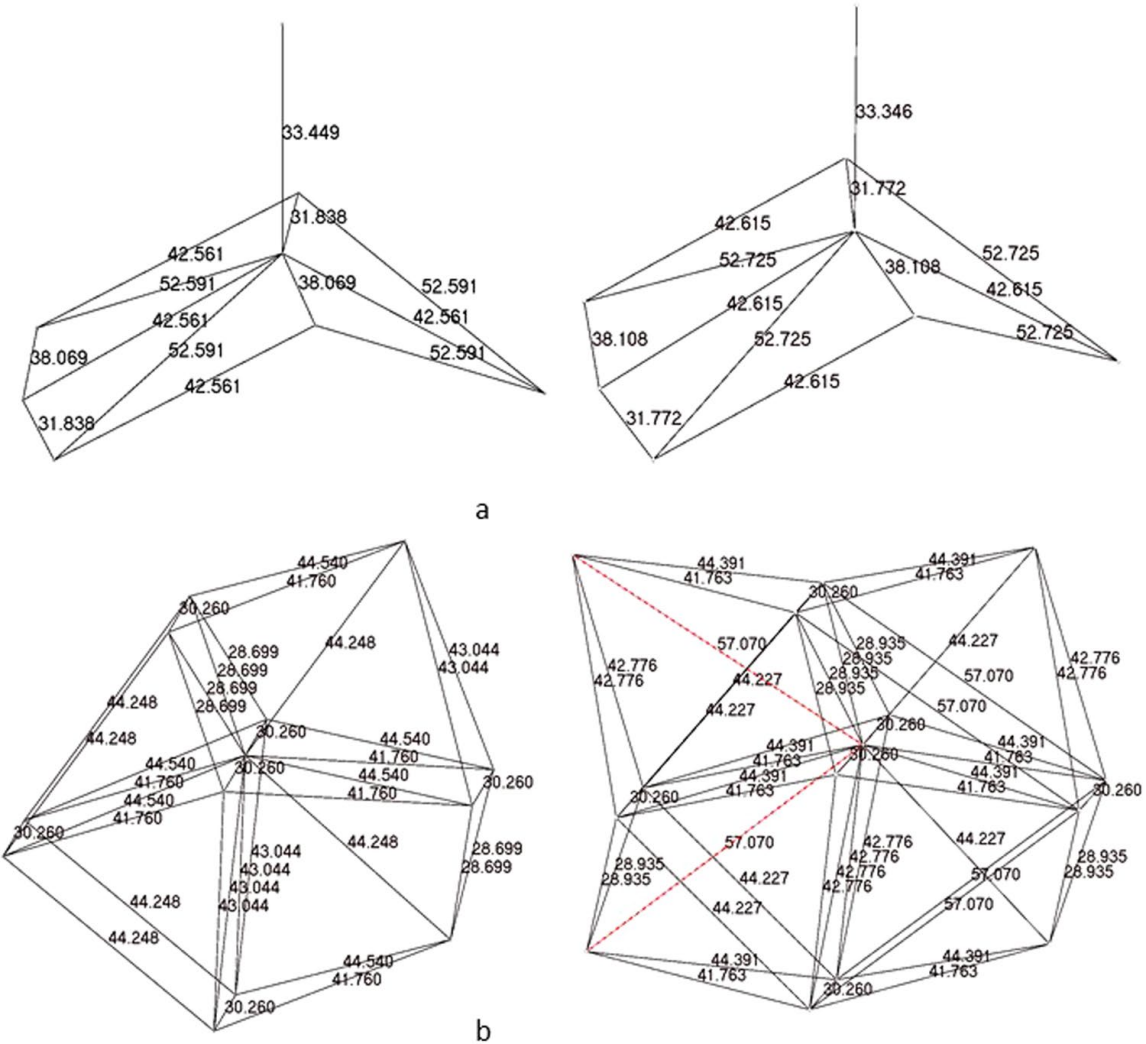
Underlying nets of crystal structures of (**a**) 4a3X^52^ and (**b**) 2xvs^53^ determined by ‘Distance’ (left) and ‘Domains’ (right) methods. The additional weak contacts, which are determined by the ‘Domains’ method, are shown by red lines. Distances between the molecular centers of mass are given in Å.

Since packing contacts were determined with two different approaches in protein crystals (‘Distance method’, see Methods) and in small molecule crystals (‘Domains method’), it is mandatory to compare the results obtained by following these two different approaches and to verify that they produce identical results. For this purpose we have computed intermolecular contacts for some proteins with the ‘Domains method’. In most cases, the results of both methods coincide (Fig. 5a), however, even if they differ, this concerns only weak long contacts, which the ‘Domains’ method can consider in contrast to the ‘Distance’ method (Fig. 5b). Since the weak contacts are not considered in the multilevel topological analysis, the topologies obtained by the two methods can be assumed to be comparable.

Table 1 shows the 20 most frequent underlying nets for monomeric protein crystals as well as for small molecule crystals. One can see that small molecules tend to dense packings with coordination numbers ranging from 12 to 14, in agreement with previous results^27,28^. On the contrary, coordination numbers are very often much lower in protein crystals, in agreement with the fact that a considerable fraction of their volume is occupied by liquid water. However, all underlying nets of protein crystals in Table 1 are well known and almost all of them (except nets **chb**, **smt**, **yfh**) are **fcu** and **hcp** subnets. Interestingly, the 14-coordinated body-centered cubic packing (**bcu-x**), which is the most frequent in small molecule crystals is rather infrequent in protein crystals (Table 1). Thus protein crystals follow the close packing topologies but with some gaps, which are obviously filled by the solvent molecules and decrease coordination numbers of protein molecules.

**Table 1:** Most frequent underlying net topologies in crystals of monomeric and dimeric proteins and of small molecules.

Monomeric proteins			Dimeric proteins			Small molecules		
Underlying net	CN	N° (%)	Underlying net	CN	N° (%)	Underlying net	CN	N° (%)
**fcu**	12	23 (5.8)	**fcu**	12	15 (7.2)	**bcu-x**	14	31815 (30.2)
**bct**	10	19 (4.8)	**hex**	8	14 (6.8)	**tcg-x**	14	12734 (12.1)
**hex**	8	16 (4.1)	**hcp**	12	11 (5.3)	14T3	14	12475 (11.8)
**vcs**	8	16 (4.1)	**bct**	10	8 (3.9)	**gpu-x**	14	11355 (10.8)
**sxd**	6	12 (3.0)	**cco**	10	8 (3.9)	**fcu**	12	5438 (5.2)
**hcp**	12	12 (3.0)	**ecu**	8	8 (3.9)	14T8	14	3694 (3.5)
**feb**	10	12 (3.0)	**feb**	10	6 (2.9)	**bcu-x**-13-C2/m	13	2806 (2.7)
**chb** ^†^	10	10 (2.5)	**pcu**	6	5 (2.4)	14T10	14	2328 (2.2)
**eca**	8	10 (2.5)	**bcu-x**	14	5 (2.4)	**hcp**	12	2073 (2.0)
**lcy**	6	9 (2.3)	**bcu**	8	4 (1.9)	14T5	14	1788 (1.7)
**ose**	7	9 (2.3)	**sve**	7	3 (1.4)	14T9	14	1256 (1.2)
**yfh** ^†^	5	9 (2.3)	**ose**	7	3 (1.4)	14T24	14	1215 (1.2)
**pcu**	6	8 (2.0)	**eca**	8	3 (1.4)	14T6	14	1091 (1.0)
**tcg**	10	8 (2.0)	**sxd**	6	3 (1.4)	14T18	14	1076 (1.0)
**cco**	10	7 (1.8)	**bnn**	5	3 (1.4)	**bcu-x**-13-P21/c	13	884 (0.8)
**dia**	4	6 (1.5)	**bsn**	6	2 (1.0)	13T3	13	850 (0.8)
**ecu**	8	5 (1.3)	**tcg-x**	14	2 (1.0)	13T4	13	717 (0.7)
**acs**	6	4 (1.0)	**wnj**	7	2 (1.0)	13T5	13	680 (0.6)
**smt** ^‡^	6	4 (1.0)	14T3	14	2 (1.0)	**bcu-x**-13-Cmca	13	661 (0.6)
**bcu-x** ^‡^	14	4 (1.0)	Other topologies	—	11 (5.3)	14T37	14	500 (0.5)

^†^Subnet only of the **fcu** net. ^‡^Neither **fcu** nor **hcp** subnet.

It is interesting to observe that while few topologies are extremely more frequent than others in small molecule crystals, this is not observed in protein crystals. 30% of the small molecule crystals are associated with the most frequent topology (**bcu-x**) and 65% of them have one of the four most common topologies (**bcu-x**, **tcg-x**, 14T3, **gpu-x**). On the contrary, only 6% of the protein crystals are associated with the most frequent topology (**fcu**) and only 19% of them have one of the four most common topologies (**fcu**, **bct**, **hex**, **vcs**). This suggests that the suboptimal packing of the protein molecules in the crystal state allows a wider number of topologies and none of them can be much more frequent than the others.

Similar results are observed for the crystals of dimeric proteins where there is one dimer per asymmetric unit (Table 1) and for the small molecule crystals where there are two independent molecules in the asymmetric unit (Table 2). On the contrary, the underlying nets of crystals of monomeric proteins where the asymmetric unit contains two independent molecules set are very diverse and it was impossible to separate any preferable motif (Table 2).

**Table 2:** Most frequent underlying net topologies in crystals of protein and small molecules with two molecules in the asymmetric unit.

Small molecules			Proteins		
Underlying net	CN	N° (%)	Underlying net	CN	N° (%)
**bcu-x**	14	1846 (18.0)	**hcp**	12	3 (1.8)
**gpu-x**	14	976 (9.5)	**nce**	9	2 (1.2)
14T3	14	707 (6.9)	**ose**	7	2 (1.2)
**tcg-x**	14	550 (5.4)	9,9T5	9	2 (1.2)
**bcu-x**-13-C2/m	13	268 (2.6)	**pcu**	6	2 (1.2)
14T5	14	200 (2.0)	**fcu**	12	2 (1.2)
14T8	14	194 (1.9)	**srs**	3	2 (1.2)
**fcu**	12	193 (1.9)	**sxd**	6	2 (1.2)
14T10	14	187 (1.8)	**hex**	8	2 (1.2)
14T6	14	163 (1.6)	**chb**	10	2 (1.2)
12,14T18	12 14	117 (1.1)	**oob**	7	1 (0.6)
14T9	14	114 (1.1)	**wkx**	5	1 (0.6)
14T37	14	93 (0.9)	**tcj**	10	1 (0.6)
**bcu-x**-13-Cmca	13	87 (0.8)	12T2171	12	1 (0.6)
14,14T47	14	82 (0.8)	Other topologies	—	44 (26.8)

### Local topologies: the coordination numbers

**Figure 6:**
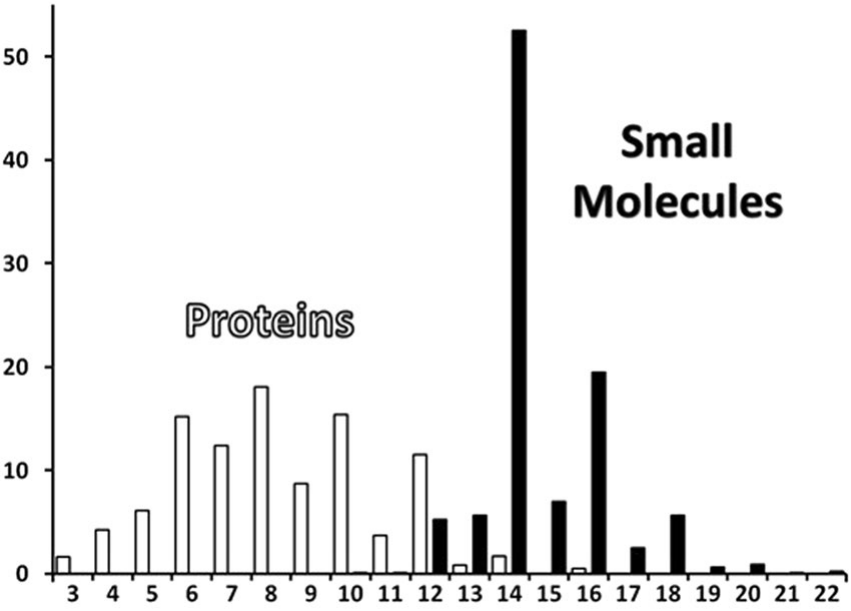
Distribution of the coordination numbers (%) for all the structures of proteins examined (see full data in Table S2) compared to the CN for small molecules.

Here the attention is focused on the coordination numbers, independently of the overall topology. This simple analysis of the individual coordination numbers shows a considerable difference between protein crystals and small molecule crystals. The most frequent coordination numbers for protein molecules are 7 and 8, while small molecules prefer higher coordination numbers equal to or larger than 14 (Fig. 6; see also supplementary Table S2).

Like for the overall topology analysis, we see that the most frequent coordination number of small molecules (14) is observed in a large fraction of crystals (52%) while the most frequent coordination number for proteins (7 or 8) is observed only in a smaller fraction of crystals (about 20%). Moreover, small molecules can adopt 25 different coordination number values (from 4 to 32) while proteins can have only 14 different coordination number values (ranging from 1 to 16 – coordination numbers 2 and 15 are never observed).

All these observations point out that proteins, which are suboptimally packed in their crystals, can hardly by surrounded by numerous other proteins and that the palette of their coordination numbers is considerably more limited.

### Rare overall topologies

Although the overall topologies are quite similar in protein and in small molecule crystals, protein molecules can form packings of a topology that never occurs in packings of small molecules. Among 394 monomeric and 207 dimeric protein structures there are 31 and 10 underlying nets, respectively, which were not found in the packings of small molecules, and all of them have a unique topology (see Table S3 in the Supplementary Material). Among the crystal structures with two independent protein molecules per asymmetric unit, there are 44, which have unique overall topologies never revealed in the small molecule crystals (Table 2).

This means that from 5 to 26% of protein molecules essentially differ from small molecules by their ability to be packed.

### Multilevel topological analysis

In order to reach a better understanding of the differences between the topologies of protein and small molecule crystals, we performed the multilevel analysis for the molecular packings in 394 monomeric protein structures and 105,549 structures of small molecules. As a result, 1,799 and 1,144,539 structure representations were generated, respectively.

**Figure 7:**
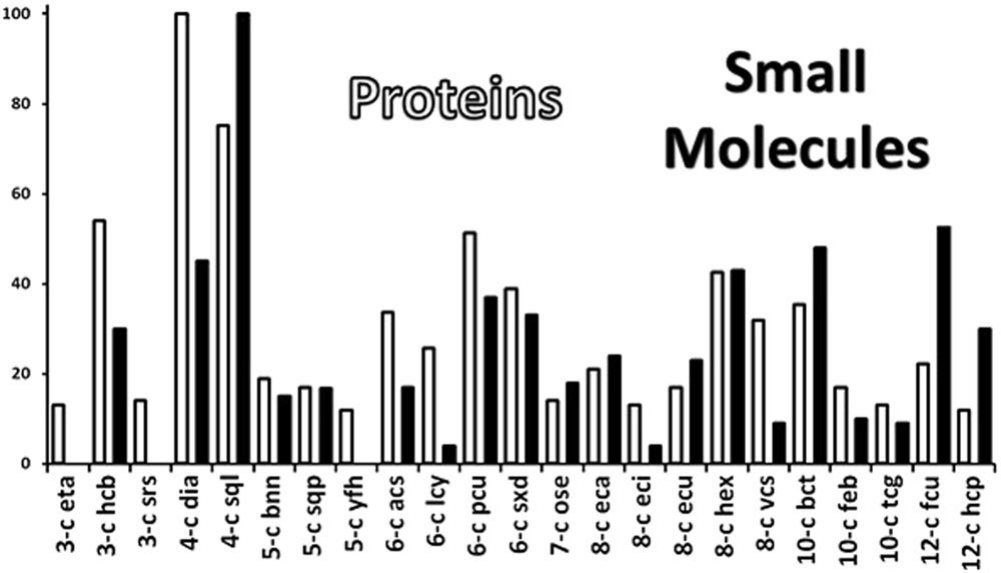
Distribution of the underlying nets observed in the multilevel topological analysis of the proteins and small molecule crystals ranked by CN and scaled to 100 on the most frequent in each set (**dia** for proteins and layers **sql** for small molecules).

The underlying net topologies are summarized in Table S4 (see Supplementary Material). In general, the underlying motifs that are frequent in protein crystals are also frequent in small molecules crystals. The frequency of the protein topologies and the frequency of the small molecule topologies shown in Table S4 correlate well (Pearson correlation coefficient = 0.625; Spearman correlation coefficient = 0.563) as shown in the histogram in Fig. 7.

**Figure 8:**
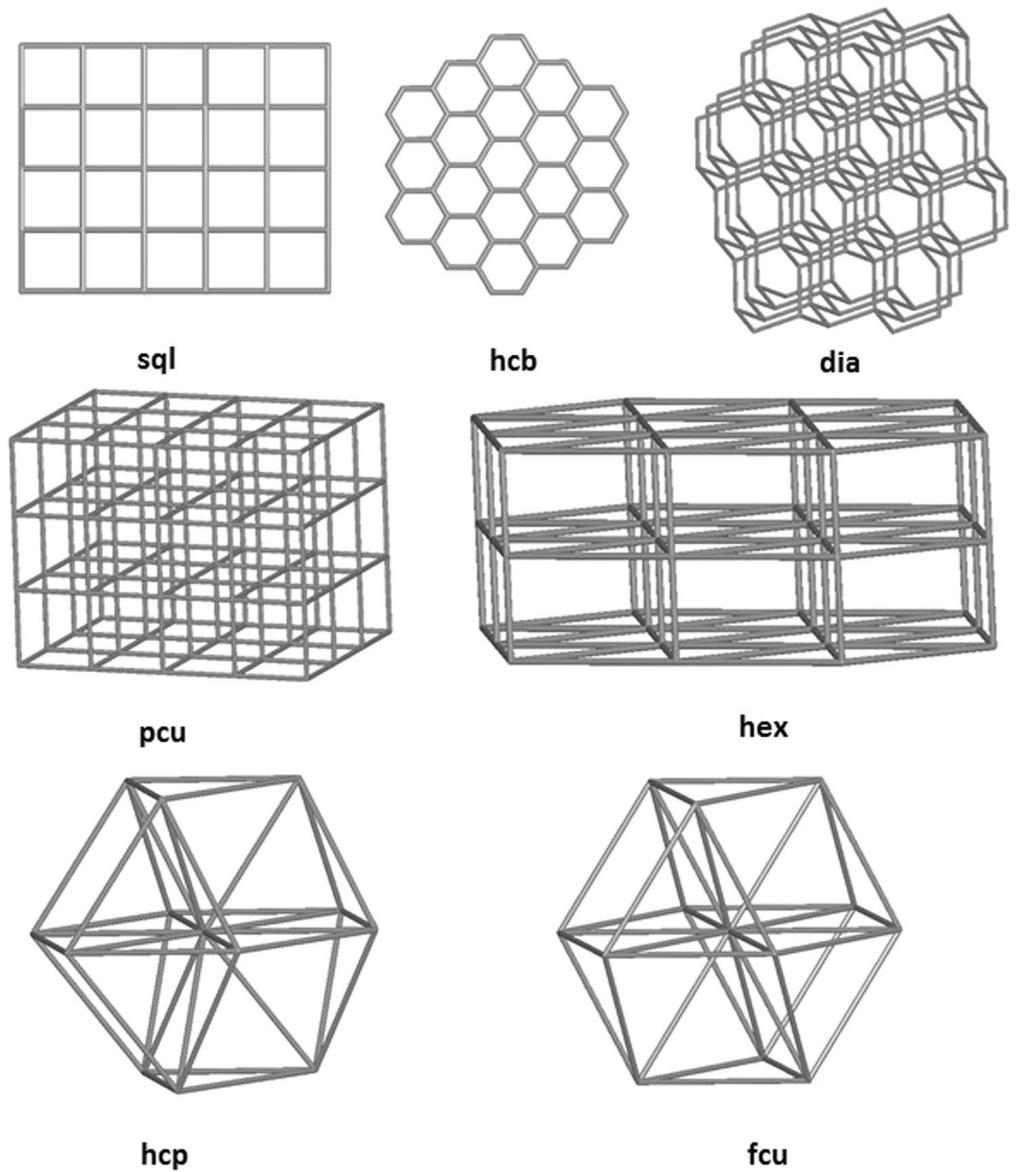
Most typical underlying motifs in packings of protein and small organic molecules.

It appears that the underlying motifs of packings in both protein and small molecule crystals have simple and quite common topologies, such as **sql**, **hcb**, **dia**, **pcu**, **hex** (Fig. 8). The role of close-packed motifs (**fcu**, **hcp**, Fig. 8) is expectedly higher for small molecules than for macromolecules. Since the multilevel analysis reveals the basic motifs (*skeletons*), which are formed by the strongest intermolecular contacts, the results obtained mean that that the skeletons in molecular crystals are mostly independent of the size and shape of the molecule, while the total packing can vary in proteins compared to small molecules.

### Topology *versus* thermodynamics

To find correlations between thermodynamic parameters and molecular connectivity we examined all the 394 protein structures of the *monomer* set. A total of 3,156 crystal packing contacts were identified. We observed that the number of intermolecular contacts (*NC*) is strictly related to the *ΔG*_*diss*_. PISA does not compute the number of intermolecular contacts but computes the buried solvent area (*BSA*; the patch of solvent accessible surface of molecules A and B that become inaccessible to the solvent because of the interaction between A and B). There is a strict relationship between *NC* and *BSA*:

3 $$BSA=6.66\cdot NC$$

with Pearson’s correlation coefficient of 0.945. This is obviously not surprising and it means that a single intermolecular contact buries nearly 7 Å^2^ of solvent accessible surface. This means that about 30% of the surface of an atom of radius equal to 1.4 Å is buriable in a protein-protein interface, while the rest is buried intra-molecularly. Also the relationship between BSA and *ΔG*_*diss*_ is strict:

4 $${\rm{\Delta }}{G}_{diss}=-11.56+0.00036\cdot BS{A}^{1.36}$$

with Pearson’s correlation coefficient of 0.793. We note that this is a mere mathematical fit of the data, which has no physico-chemical meaning. Consequently, it is possible to fit the dependence of *ΔG*_*diss*_ on *NC* as:

5 $${\rm{\Delta }}{G}_{diss}=-11.25+0.116{(0.123\cdot NC)}^{1.24}$$

with Pearson’s correlation coefficient of 0.770. The average absolute difference between the *ΔG*_*diss*_ values computed by PISA and those computed on the basis of the *NC* values is only 2.00 (standard error 0.03) kcal/mol. We observe that the |*ΔG*_*diss*_ - *NC* | values tend to increase slightly with *NC*. The average |*ΔG*_*diss*_ - *NC* | values are only 1.46 (0.03) kcal/mol when 1 ≤ *NC* ≤ 30 (808 observations) and they growth to 3.6(0.3) kcal/mol when 210 ≤ *NC* ≤ 240 (74 observations). For larger *NC* values, there are too few observations to make reasonable statistics. Although this trend indicates that the fitting model is statistically inappropriate, for practical reasons it is sufficient to estimate *ΔG*_*diss*_ values with an average error of about 2–3 kcal/mol in most cases.

## Conclusions

The topologies of carefully selected high-quality protein crystal structures have been determined and compared to those of a large set of small molecule crystal structures. It is known that protein crystals are suboptimally packed, since they contain channels filled with liquid water. Therefore, the coordination numbers in protein structures are considerably lower than in small molecule crystals. Surprisingly, however, we have found their topologies very similar. Although it is impossible, based on the data presented in this communication, to provide a strict and definitive explanation for this surprising similarity, it is clear that some molecular mechanism must exist. It is possible to hypothesize, for example, that during the early stages of nucleation, only relatively few types of assemblies, with a specific stereochemistry, are sufficiently stable to be able to accumulate and to continue to grow into real crystals. At this regard, it might be interesting to remember that three main differences exist between protein and small molecule crystallization^41–44^. First, the kinetics of protein crystal nucleation and growth are, in general, two or three orders of magnitude slower than for small molecules, because of the protein larger size and lowered diffusivity^45,46^. Second, protein crystals nucleation occurs at very high levels of supersaturation, often two or three orders of magnitude greater than that required to sustain crystal growth. Third, proteins may assume several distinctive solid states that include amorphous precipitates, oils or gels as well as crystals, and most of these are kinetically favored. Further studies are necessary and additional data must be considered to find out the rationale why topology seems to be independent of packing efficiency and crystallization.

## Electronic supplementary material

Supplementary information
