# Supplementary material for: Packing topology in crystals of proteins and small molecules: a comparison
Source: Sci Rep. 2017 Oct 16;7:13209. doi: 10.1038/s41598-017-12699-4 (PMC5643379; doi:10.1038/s41598-017-12699-4)
Supplement: Supplementary file 1 — Supplementary information [file 41598_2017_12699_MOESM1_ESM.pdf]

Packing topology in crystals of proteins and small molecules: a comparison

Oliviero Carugo,[a,b,*] Olga A. Blatova,[c] Elena O. Medrish,[c] Vladislav A. Blatov,[c,d,*] Davide M. Proserpio[c,e,*]

[a] Department of Chemistry, University of Pavia, viale Taramelli 12, I-27100, Pavia, Italy

[b] Department of Structural and Computational Biology, University of Vienna, Campus Vienna Biocenter 5, A-1030 Vienna, Austria

[c] Samara Center for Theoretical Materials Science (SCTMS), Samara University, Ac. Pavlov St. 1, Samara 443011, Russia.

[d] School of Materials Science and Engineering, Northwestern Polytechnical University, Xi'an, Shaanxi 710072, People's Republic of China.

[e] Università degli Studi di Milano, Dipartimento di Chimica, Via C. Golgi 19, 20133 Milano, Italy.

Supplementary Information

Supplemetary Table S1. Lists of the identification codes of the structures of the Protein Data Bank.

monomer set

1a7d 1ac5 1bhe 1bs1 1c44 1eh5 1eq6 1ew4 1eyh 1f5b 1fl2 1fnf 1fob 1fw9 1gq8 1h2e 1h6h 1hk0 1hq0 1i60 1iee 1ijb 1io2 1j1g 1jaw 1jfx 1jmm 1jwq 1k12 1kcq 1kfr 1ks8 1lj5 1lva 1m40 1m6t 1mid 1mix 1mk0 1mxg 1nb9 1ne9 1nep 1nte 1obv 1olr 1p2x 1p3c 1pa7 1pxu 1q2y 1qnx 1rc9 1rlw 1ruw 1rw1 1s31 1sdj 1ssx 1t71 1tke 1tov 1u6t 1ua7 1uas 1ukf 1uoy 1ux7 1vgj 1wd3 1whz 1wol 1wq3 1wwi 1wza 1x3k 1x46 1x8q 1xeu 1xw3 1y57 1yn4 1yp5 1yqe 1yt3 1yu5 1yzf 1z6m 1z77 1zeq 1zhv 1zl7 1zt3 1zx5 2a7b 2a98 2ams 2ayd 2azp 2b4l 2bk8 2c0h 2c3g 2cd7 2cg7 2cu9 2cvj 2cw4 2cwy 2cyg 2cyj 2cyy 2d3y 2d48 2d58 2d5b 2dt8 2dyi 2ehg 2ej9 2erf 2esk 2evb 2eyi 2f82 2fba 2fbo 2fj9 2fjz 2fph 2frg 2fuz 2fy6 2g5x 2g6f 2gg2 2ggq 2gh4 2gke 2gpi 2gsj 2gy5 2hc8 2hd9 2he7 2hpj 2hq0 2i4a 2i6v 2in0 2iqy 2is9 2j9v 2jg6 2jhy 2jlq 2ns02nsc 2nsz 2nvg 2o31 2ocg 2of3 2oit 2okt 2ovj 2ovu 2oyp 2p8v 2pes 2pet 2pv4 2q88 2q9v 2qdx 2qeh 2qen 2qht 2qjl 2qmt 2qnk 2qsk 2qzq 2r0s 2r1n 2rbk 2rj2 2rkq 2uux 2ux7 2v14 2v3i 2v6g 2v9v 2vac 2vfk 2vhk 2w0i 2w39 2w5q 2w86 2wbx 2wfb 2wiy 2x49 2x5o 2x8x 2x9z 2xhj 2xjk 2xjp 2xoe 2xu9 2xvs 2y44 2y7b 2z62 2znr 2zu5 2zzj 3a0t 3a4c 3a5e 3aap 3ab6 3acp 3ahw 3akb 3akq 3b0t 3b35 3b92 3bod 3c7f 3c7x 3cjj 3clm 3ct5 3cu9 3cz7 3d30 3d3y 3dan 3db7 3deo 3dha 3dj9 3dnz 3dqy 3e4g 3e8v 3e9v 3edh 3f19 3f45 3f7w 3fgh 3fhg 3fk5 3foj 3fp5 3fwa 3g5t 3g7m 3gmv 3go5 3h0o 3h31 3hb2 3hbn 3hfw 3hxw 3iaj 3ils 3ip0 3iqb 3iv4 3jtm 3jz9 3k6i 3kt9 3kvd 3l4h 3l5i 3l6g 3lp5 3lwx 3m5q 3m70 3mao 3mgw 3mp2 3mwq 3mwz 3mx7 3n35 3n6t 3no3 3o04 3o3x 3o5p 3od3 3onj 3ooi 3oos 3ozu 3p06 3p6l 3pdd 3pm2 3psa 3pvh 3px8 3pxl3pyc 3q1n 3r72 3rgq 3rja 3rjp 3s0e 3s9x 3sbm 3tbn 3tip 3trd 3tt9 3uci 3ue2 3ufc 3ui5 3vfi 3vnw 3vpy 3vtg 3w2g 3w43 3wcq 3wdn 3web 3wjt 3zfp 3zl2 3zuc 3zud 3zyp 4a3x 4a4j 4ac1 4ale 4awn 4az6 4b6w 4bgc 4byz 4c7g 4cfy 4dpb 4dvc 4e7g 4esp 4esq 4f55 4gn2 4gqr 4hh5 4hyq 4i8g 4inw 4itm 4iye 4j5e 4j5q 4jm1 4jp6 4kmy 4lbs 4ld6 4lmp 4m1v 4mb7 4mrh 4mxd 4n19 4n6x 4nli 4nuh 4nvb 4o1r 4o7q 4orl 4p3x 4p5s 4ps6

dimer set

1a8u 1b7g 1b8a 1dow 1eej 1ejd 1fyf 1g29 1gu7 1gyy 1i88 1itu 1iu8 1iyb 1jlt 1k7h 1kzk 1lqa 1lwd 1miz 1nme 1ntv 1oai 1ofz 1p4k 1qh5 1r0r 1r8o 1rg0 1rku 1scj 1sj1 1t06 1t1v 1to2 1u0s 1u6r 1ugp 1usc 1utx 1vc1 1vc4 1w9a 1wkr 1y89 1y9w 1yrk 1zuy 1zzg 2aa4 2alg 2axw 2b9d 2bkr 2blf 2c1l 2cki 2cvi 2dct 2dfi 2dql 2dsj 2dvn 2e5y 2ecu 2ei5 2ek0 2fae 2fff 2g6t 2gs9 2gsk 2gty 2hal 2hdb 2hp4 2hpl 2i5g 2iq6 2iyc 2jgz 2jhf 2o9v 2od4 2omz 2or2 2ori 2ou5 2oya 2p53 2p62 2p97 2p9h 2pv1 2qgi 2qiw 2qos 2r1u 2vg0 2vkj 2vn6 2wlv 2wqw 2x2w 2xr4 2xxn 2y5f 2ywl 2z30 2z5e 2z64 2z7f 2zdp 2zx2 3a3d 3a6q 3b90 3bhl 3cnk 3czz 3dmc 3e2c 3ent 3erx 3fap 3fas 3ff5 3fju 3fou 3g3s 3g4e 3gae 3ghd 3gmx 3h6r 3hbu 3hcn 3hdb 3hh7 3icy 3id3 3ifj 3k1r 3ke0 3las 3lb2 3liq 3m7u 3mbk 3mhr 3mjo 3mmh 3mwj 3n40 3nbc 3ngh 3ooo3oq3 3p72 3p73 3pt8 3pwf 3qdx 3qrb 3tfj 3u1y 3u2u 3u4z 3umo 3uvw 3vay 3via 3vrg 3w0e 4a7u 4alo 4auy 4avr 4b8y 4bi3 4bk6 4dzz 4eir 4eq7 4eqs 4esw 4g5a 4gn4 4gr7 4gvc 4hfq 4hia 4hke 4hvw 4ics 4ifk 4ij5 4iwt 4jh2 4jqt 4jtm 4jzx 4kem 4m5s 4mjd 4nq1 4oo4

double set

1bir 1c7f 1cqx 1dk7 1dwu 1ece 1ejd 1fsl 1g1b 1gzj 1hjz 1idj 1ioo 1k7h 1kh0 1ljp 1ncn 1nyc 1obo 1ogs 1oil 1oio 1oj4 1pe9 1pva 1pvs 1pxz 1qgj 1qmd 1s5v 1t2b 1tgr 1tvf 1usi 1uxz 1v6s 1vav 1vc4 1vcd 1vcl 1xgn 1xm8 1xwv 1yix 1yn3 2ahf 2atb 2bkm 2bv2 2bwr 2bxx 2cxd 2d42 2dsn 2fae 2gax 2ggs 2gte 2hba 2hds 2hyw 2idr 2j45 2j5y 2jg5 2jkw 2ntp 2nw0 2o6s 2oya 2p6h 2pr0 2pr7 2psp 2qb1 2qeb 2qqr 2qt6 2qy1 2r2q 2ri9 2rjw 2v33 2vlw 2w87 2x0k 2yoy 2z8q 2zd2 2zqo 3ab5 3aub 3b90 3bh4 3boh 3c7m 3cjk 3cql 3cvb 3d78 3d95 3e11 3ejw 3f9r 3fdo 3ffv 3fid 3fvk 3g4e 3g98 3gae 3gmy 3h63 3h6r 3hdb 3hnk 3hy0 3ifj 3k5r 3k6f 3l01 3l50 3mvi 3nwp 3o1f 3obl 3ogn 3omd 3oyo 3qhb 3qqi 3qry 3rj6 3s0g 3sim 3u25 3u4z 3u8i 3v8i 3via 3w40 3w9v 3zk9 3zl1 4au9 4bi4 4c1s 4dok 4dwq 4dzz 4e1y 4eir 4eq7 4f8k 4gcn 4ger 4gr74hs5 4iyb 4j4b 4jp7 4k1x 4m1r 4oo4

Supplementary Table S2. Most frequent coordination numbers (CN) in protein and small molecule crystals.

Monomeric proteins		Dimeric proteins		Proteins with two molecules in cell		Small molecules	
CN	N° (%)	CN	N° (%)	CN	N° (%)	CN	N° (%)
8	78 (19.8)	8	44 (21.3)	7	59 (18.0)	14	87272 (52.5)
10	72 (18.3)	10	41 (19.8)	9	49 (14.9)	16	32404 (19.5)
6	70 (17.7)	12	34 (16.4)	6	47 (14.3)	15	11684 (7.0)
12	46 (11.7)	6	24 (11.6)	8	46 (14.0)	18	9275 (5.6)
7	39 (9.9)	7	17 (8.2)	10	30 (9.1)	13	9259 (5.6)
5	26 (6.6)	9	12 (5.8)	12	27 (8.2)	12	8435 (5.1)
4	21 (5.3)	5	9 (4.3)	5	22 (6.7)	17	4165 (2.5)
9	20 (5.1)	14	9 (4.3)	11	21 (6.4)	20	1529 (0.9)
11	8 (2.0)	4	8 (3.9)	3	11 (3.4)	19	997 (0.6)
14	6 (1.5)	11	5 (2.4)	4	10 (3.0)	22	362 (0.2)
3	4 (1.0)	16	2 (1.0)	13	4 (1.2)	10	240 (0.1)
16	3 (0.8)	13	2 (1.0)	14	1 (0.3)	11	237 (0.1)
13	1 (0.3)			1	1 (0.3)	21	175 (0.1)
						24	71
						8	40
						23	32
						9	31
						26	30
						6	16
						25	12
						28	8
						32	2
						7	2
						4	2
						30	2

Supplementary Table S3. The underlying nets for packings of protein crystals with rare and unique topology not observed in small molecule crystals.

Name	CN	Topology
**Monomers**		
1u6t	16	16T6
3uci	16	16T7
3ue2	16	16T8
3acp	11	11T1583
3go5	11	11T1584
4iye	11	11T1585
1eyh	10	10T1528
2nsc	10	10T1529
2x8x	10	10T1530
3dan	10	10T1531
1iee	9	9T904
2fj9	9	9T905
2xhj	9	9T906
3g7m	9	9T907
3jz9	9	9T908
2cyy	8	8T762
3zuc	8	8T763
1tov	7	7T333
1y57	7	7T334
2bk8	7	7T335
2c3g	7	7T336
2ux7	7	7T337
3b92	7	7T338
3dnz	7	7T339
3fhg	7	7T340
3mwz	7	7T341
4lmp	7	7T342
1jmm	6	6T226
3aap	6	6T227
3b35	6	6T228
3p06	6	6T229

**Dimers**		
2omz	16	16T9
2hp4	10	10T1535
2p53	10	10T1536
4auy	10	10T1537
2x2w	9	9T911
3umo	9	9T912
4gr7	9	9T913
2wqw	8	8T775
1to2	7	7T347
2alg	7	7T348

Supplementary Table S4. Underlying nets obtained by multilevel analysis of protein and small molecule crystal structures. For each underlying net the following quantities are given: the coordination number (CN), the number of occurrences in monomeric protein crystals (proteins) and the number of occurrences in small molecule crystals (small molecules). Nets are ranked according to their frequency in protein crystals.

Underlying net	CN	proteins	small molecules
**dia**	4	113	34340
**sql**	4	85	75561
**hcb**	3	61	22666
**pcu**	6	58	28327
**hex**	8	48	32560
**sxd**	6	44	24867
**bct**	10	40	36383
**acs**	6	38	12725
**vcs**	8	36	7076
**lcy**	6	29	3030
**fcu**	12	25	47144
**eca**	8	24	18411
**bnn**	5	22	11702
**ecu**	8	19	17393
**sqp**	5	19	12758
**feb**	10	19	7681
**srs**	3	16	81
**ose**	7	16	13558
**eci**	8	15	3243
**tcg**	10	15	6430
**eta**	3	15	10
**hcp**	12	14	22666
**yfh**	5	14	35
**chb**	10	13	16835
**bsn**	6	13	4912
**cco**	10	11	14519
**hxl**	6	11	34139
**ths**	3	10	998
